# Altered Brain Cholesterol Machinery in a Down Syndrome Mouse Model: A Possible Common Feature with Alzheimer’s Disease

**DOI:** 10.3390/antiox13040435

**Published:** 2024-04-03

**Authors:** Erica Staurenghi, Gabriella Testa, Valerio Leoni, Rebecca Cecci, Lucrezia Floro, Serena Giannelli, Eugenio Barone, Marzia Perluigi, Gabriella Leonarduzzi, Barbara Sottero, Paola Gamba

**Affiliations:** 1 Department of Clinical and Biological Sciences, University of Turin, San Luigi Hospital, 10043 Orbassano, Italy; erica.staurenghi@unito.it (E.S.); rebecca.cecci@unito.it (R.C.); lucrezia.floro@unito.it (L.F.); serena.giannelli@unito.it (S.G.); gabriella.leonarduzzi@unito.it (G.L.); barbara.sottero@unito.it (B.S.); paola.gamba@unito.it (P.G.); 2 Laboratory of Clinical Pathology, Hospital Pio XI of Desio, ASST-Brianza and Department of Medicine and Surgery, University of Milano-Bicocca, 20832 Desio, Italy; valerio.leoni@unimib.it; 3 Department of Biochemical Sciences “A. Rossi-Fanelli”, Sapienza University, 00185 Roma, Italy; eugenio.barone@uniroma1.it (E.B.); marzia.perluigi@uniroma1.it (M.P.)

**Keywords:** Down syndrome, Alzheimer’s disease, cholesterol, oxysterols, neuroinflammation, oxidative stress, DHCR24, HMGCR, CYP46A1

## Abstract

Down syndrome (DS) is a complex chromosomal disorder considered as a genetically determined form of Alzheimer’s disease (AD). Maintenance of brain cholesterol homeostasis is essential for brain functioning and development, and its dysregulation is associated with AD neuroinflammation and oxidative damage. Brain cholesterol imbalances also likely occur in DS, concurring with the precocious AD-like neurodegeneration. In this pilot study, we analyzed, in the brain of the Ts2Cje (Ts2) mouse model of DS, the expression of genes encoding key enzymes involved in cholesterol metabolism and of the levels of cholesterol and its main precursors and products of its metabolism (i.e., oxysterols). The results showed, in Ts2 mice compared to euploid mice, the downregulation of the transcription of the genes encoding the enzymes 3-hydroxy-3-methylglutaryl-CoA reductase and 24-dehydrocholesterol reductase, the latter originally recognized as an indicator of AD, and the consequent reduction in total cholesterol levels. Moreover, the expression of genes encoding enzymes responsible for brain cholesterol oxidation and the amounts of the resulting oxysterols were modified in Ts2 mouse brains, and the levels of cholesterol autoxidation products were increased, suggesting an exacerbation of cerebral oxidative stress. We also observed an enhanced inflammatory response in Ts2 mice, underlined by the upregulation of the transcription of the genes encoding for α-interferon and interleukin-6, two cytokines whose synthesis is increased in the brains of AD patients. Overall, these results suggest that DS and AD brains share cholesterol cycle derangements and altered oxysterol levels, which may contribute to the oxidative and inflammatory events involved in both diseases.

## 1. Introduction

Down syndrome (DS), or trisomy 21, is a complex and highly variable chromosomal disorder that affects one in 650–1000 newborns globally. It is indicated as a genetically determined form of Alzheimer’s disease (AD), a progressive neurodegenerative illness whose etiology is not yet fully understood, since DS subjects are extremely predisposed to early-onset AD. Due to the increased life expectancy of the DS population, the prevalence of AD comorbidity has increased, with AD becoming the primary cause of death of DS individuals [1].

Both AD and DS individuals with AD share similar neuropathological and clinical features, including the formation of amyloid β (Aβ) plaques and neurofibrillary tangles of hyperphosphorylated Tau (pTau), which precociously accumulate in individuals with DS, as well as early symptoms of dementia. These hallmarks are ascribed to the presence, on human chromosome 21, of genes specifically related to AD etiology, such as amyloid precursor protein (*APP*) and β-secretase (*BACE2*), although the aberrant expression of other chromosome 21 coding/noncoding genes may also contribute [2,3,4]. In addition, other events common to both pathologies, in particular the exacerbation of oxidative and inflammatory responses, participate in AD-like cerebral damage in DS subjects [5,6,7].

**Figure 1:**
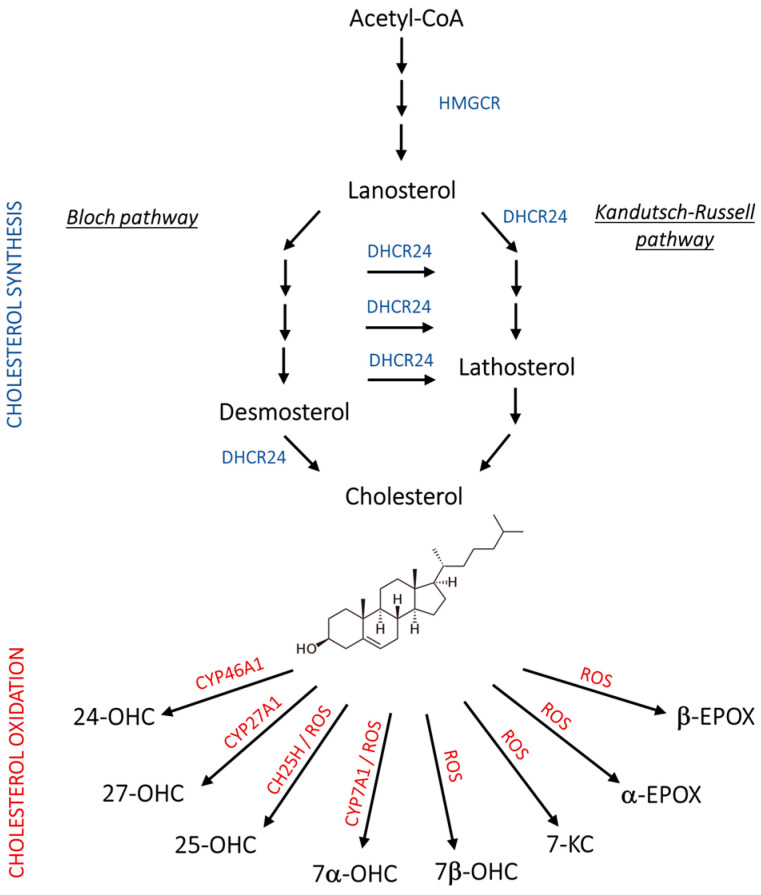
Schematic representation of cholesterol synthesis and oxidation. 24-OHC, 24(S)-hydroxycholesterol; 25-OHC, 25-hydroxycholesterol; 27-OHC, 27-hydroxycholesterol; 7α-OHC, 7α-hydroxycholesterol; 7β-OHC, 7β-hydroxycholesterol; 7-KC, 7-ketocholesterol; Acetyl-CoA, acetyl coenzyme A; CH25H, cholesterol 25-hydroxylase; CYP46A1, cholesterol 24-hydroxylase; CYP27A1, cholesterol 27-hydroxylase; CYP7A1, cholesterol 7α-hydroxylase; DHCR24, 24-dehydrocholesterol reductase; HMGCR, 3-hydroxy-3-methylglutaryl-CoA reductase; ROS, reactive oxygen species.

Abnormal lipid metabolism in the brain is another key hallmark of AD that has been deeply investigated in recent years. In particular, derangements in cholesterol homeostasis have been recognized to underlie neurodegeneration, since cholesterol is fundamental for axonal growth and synaptic formation and plasticity, and its levels change with neuroinflammation [8,9]. In healthy mammals, endogenous cholesterol synthesis takes place through two main stages: (i) a “pre-lanosterol stage”, whose key player, 3-hydroxy-3-methylglutaryl-CoA reductase (HMGCR), leads to the synthesis of lanosterol starting from acetylCoA; (ii) a “post-lanosterol stage” in which cholesterol synthesis branches out into two 24-dehydrocholesterol reductase (DHCR24)-dependent alternative routes: the Bloch and the Kandutsch–Russel (K-R) pathways (Figure 1) [10,11].

Notably, cholesterol metabolism in the brain markedly differs from that in other tissues. Indeed, the impermeability of the blood–brain barrier (BBB) impedes the influx of lipoprotein-bound cholesterol from the circulation. Therefore, cholesterol supply in the brain relies almost completely on de novo synthesis. When cholesterol exceeds the cerebral cell requirement, it is enzymatically converted into more hydrophilic compounds, namely oxysterols, which are able to cross the BBB and pass into the bloodstream to be subsequently excreted by the liver as bile acids [12,13,14]. Although data about cholesterol content in the AD brain are controversial, it has been reported that altered cholesterol distribution in the plasma membrane and lipid rafts is one of the causes of dysfunctional APP processing [8].

In addition to cholesterol distribution, the accumulation of its oxidized derivatives, namely oxysterols, have been linked to AD, showing noxious or neuroprotective activities. Among the oxysterols, 24(S)- and 27-hydroxycholesterol (24-OHC and 27-OHC) arise in the brain through enzymatic reactions, and other oxysterols, such as 7-ketocholesterol (7-KC), 5α,6α-, and 5β,6β-epoxycholesterol (α-EPOX, β-EPOX), are formed non-enzymatically under pro-oxidant conditions. Moreover, 7α-hydroxycholesterol (7α-OHC) and 25-hydroxycholesterol (25-OHC) can be derived from both enzymatic and non-enzymatic reactions, as depicted in Figure 1 [13,15]. Of note, the gene encoding DHCR24, a crucial enzyme in both the post-lanosterol pathways of cholesterol synthesis (Bloch and K-R pathways), was initially identified in vulnerable brain regions of AD patients, where it was shown to be significantly downregulated as neurodegeneration progresses. Hence, its original name was seladin-1 (SELective Alzheimer’s Disease INdicator) [16]. Later, it was proven to exert, at physiological levels, a broad range of functions involved in cell survival and signaling, redox balance, and inflammation, which can explain its neuroprotective role and, consequently, the negative impact of its loss [11,14,17,18].

Of note, the apolipoprotein E-ε4 genotype (*ApoE4*), the strongest genetic risk factor for sporadic AD [19,20], is also connected to greater Aβ deposition and to earlier AD onset and premature death in DS-affected people [21,22]. In both AD and DS pathologies, *ApoE4*-dependent alterations in Aβ processing are partially due to impaired cholesterol delivery in the brain, with ApoE being responsible for cholesterol transport from astrocytes to neurons [19].

Despite its potential relevance, brain cholesterol metabolism in DS patients remains so far poorly investigated, and the data are controversial. A cholesterol decrease has been reported in the DS cerebellum and frontal cortex [23], as well as abnormal myelination in the DS brain due to a lower cholesterol content [24]. Recently, through the analysis of healthy, AD, and DS brains, cholesterol levels appeared to be increased in both AD and DS astrocyte mitochondria. Moreover, cholesterol levels were more abundant in DS hippocampal astrocyte lysosomes than in AD ones, in association with astrogliosis [25]. Nevertheless, the remarkable analogy between the AD and DS neuropathological profiles and clinical outcomes highlights the probability that these diseases share fundamental pathogenetic mechanisms underpinning their development and aggravation, and that some of them concern the cholesterol cycle and its impact on the brain inflammatory and oxidative states.

In this explorative study, we aimed at identifying the possible biochemical effectors involved in cholesterol metabolism, which could be related to the oxidative and inflammatory responses involved in the AD-like neurodegeneration typical for trisomy 21 carriers. For this purpose, we used a well-established murine model of DS, namely Ts2Cje (Ts2) mice [26,27], to unveil unknown aspects related to cholesterol metabolism in the DS brain, which in the future could provide new strategies to counteract neurodegeneration.

## 2. Materials and Methods

### 2.1. Animal Models and Brain Sample Collection

Ts2Cje (Rb(12.Ts171665Dn)2Cje) mice are a well-established murine model of DS characterized by a triple copy of a Robertsonian fusion chromosome carrying the distal end of Chr16 and Chr12. Parental generations were purchased from the Jackson Laboratories (Bar Harbour, ME, USA). The mouse colony was raised by crossbreeding Ts2 trisomic females with euploid (B6EiC3SnF1/J) F1 hybrid males (Eu). These breeding pairs produce litters containing both trisomic and euploid offspring. The pups were genotyped to determine trisomy via standard PCR using Reinoldth’s method [28]. The mice were housed in clear Plexiglas cages (20 cm × 20 cm × 20 cm) under standard laboratory conditions with a temperature of 22 ± 2 °C and 70% humidity, a 12 h light/dark cycle, and free access to food and water.

Ts2 and Eu mice, 3 and 12 months (mo.) old (n = 6/group), were sacrificed and perfused with saline, and their brain frontal cortices were dissected, flash-frozen, and stored at −80 °C. All the experiments were performed in strict compliance with the Italian National Laws (DL 116/92), the European Communities Council Directives (86/609/EEC). The experimental protocol was approved by the Italian Ministry of Health (authorization n° 522/2020-PR). All efforts were made to minimize the number of animals used in the study and their suffering.

### 2.2. Sterol Quantification Using GC-MS

Cholesterol, the cholesterol precursors (lanosterol, lathosterol, and desmosterol), and the oxysterols 7α-OHC, 7β-OHC, 24-OHC, 25-OHC, 27-OHC, 7-KC, α-EPOX, and β-EPOX were measured using isotope dilution gas chromatography–mass spectrometry (GC-MS), as described elsewhere [29].

Briefly, mouse brain samples were weighted, and a phosphate-buffered saline (PBS)–ethylenediamine tetraacetic acid (EDTA) solution (1 mg EDTA/mL PBS, Sigma-Aldrich, Merck, Darmstadt, Germany) was added to each sample in order to obtain a concentration of 100 mg tissue/mL of PBS-EDTA. Brain sample homogenates were sonicated for 15 min and then transferred to a screw-capped vial sealed with a Teflon septum together with 50 µg of epicoprostanol (Sigma-Aldrich), 500 ng of deuterated lathosterol, and 50 ng of deuterated analogues of lanosterol, 7β-OHC, 7-KC, 24-OHC, 25-OHC, and 27-OHC (Avanti Polar Lipids Inc., Birmingham, AL, USA) as internal standards, as well as 50 µL of butylated hydroxytoluene (BHT) (5 g/L, Sigma-Aldrich) and 50 µL of K3-EDTA (10 g/L, Sigma-Aldrich) to prevent autoxidation. Each vial was flushed with argon for 10 min to remove air and submitted to alkaline hydrolysis with ethanolic KOH 1M followed by two-time extraction with cyclohexane. The organic phase, separated via centrifugation and evaporated under argon, was finally converted into trimethylsilyl ethers with 100 µL of N,O-bis(trimethylsilyl)trifluoroacetamide (BSTFA) (Sigma-Aldrich).

Isotope dilution GC-MS analysis was performed using a 6890N Network GC system (Agilent Technologies, Santa Clara, CA, USA) equipped with an HP 7687 series autosampler and a HP 7683 series injector (Agilent Technologies) and coupled to a quadrupole mass selective detector HP5975B Inert MSD (Agilent Technologies). For GC separation, a B-XLB column (30 m × 0.25 mm i.d. × 0.25 µm film thickness; J&W Scientific Alltech, Folsom, CA, USA) was used. Mass spectrometric data were acquired in selected ion monitoring (SIM) mode. Peak integration was performed manually, and the analytes were quantified against internal standards using standard curves for the listed compounds.

### 2.3. RNA Extraction and Real-Time RT-PCR

The total RNA was extracted from the cortical tissues of the Ts2 and Eu mice using TRIzol™ Reagent (Invitrogen, Thermo Fisher Scientific, Waltham, MA, USA) following the manufacturers’ instructions. Possible traces of DNA were removed by using the TURBO DNA-free™ Kit (Invitrogen), and the RNA was dissolved in RNase-free water with RNase inhibitors (SUPERase-In RNase Inhibitor, Invitrogen). The amount and purity of the extracted RNA were assessed spectrophotometrically. The cDNA was synthesized via reverse transcription of 2 µg of RNA using a commercial kit and random primers (High-Capacity cDNA Reverse Transcription Kit, Applied Biosystems, Thermo Fisher Scientific).

Real-time RT-PCR was performed on 50–80 ng of the cDNA using a 7500 Fast Real-Time PCR System and TaqMan Gene Expression Assays for mouse *CYP7A1* (Mm00484150_m1), *CYP27A1* (Mm00470430_m1), *CYP46A1* (Mm00487306_m1), *CH25H* (Mm00515486_s1), *DHCR24* (Mm00519071_m1), *HMGCR* (Mm01282499_m1), *IFNA1* (Mm03030145_gH), *IFNB1* (Mm00439552_s1), *IL6* (Mm00446190_m1), *IRF5* (Mm00496477_m1), *ISG15* (Mm01705338_s1), *TNF* (Mm00443258_m1), and *GAPDH* (Mm99999915_g1) as a housekeeping gene (Applied Biosystems). The oligonucleotide sequences have not been revealed by the manufacturer due to proprietary interests. The cycling parameters were as follows: 20 s at 95 °C for AmpErase UNG activation, 3 s at 95 °C for AmpliTaq Gold DNA polymerase activation, 40 cycles of 3 s at 95 °C (melting), and 30 s at 60 °C (annealing⁄extension). The fractional cycle number (Ct) was determined for each considered gene. The gene expression results were normalized to the *GAPDH* expression levels. Relative quantification of target gene expression was achieved via a mathematical method [30].

### 2.4. Statistical Analysis

The data were analyzed using one-way ANOVAs followed by Bonferroni post hoc tests. The results were considered statistically significant when *p* < 0.05. The data are represented as means ± standard deviations (SDs). Correlation analyses were calculated using Pearson’s rank correlations and displayed as a scatter plot with a regression line to investigate the association between different pairs of variables examined in the study.

All the statistical analyses were performed using GraphPad Prism 6 software (GraphPad, La Jolla, CA, USA).

## 3. Results

### 3.1. Gene Expression of the Main Enzymes Involved in Brain Cholesterogenesis and Sterol Production

**Figure 2:**
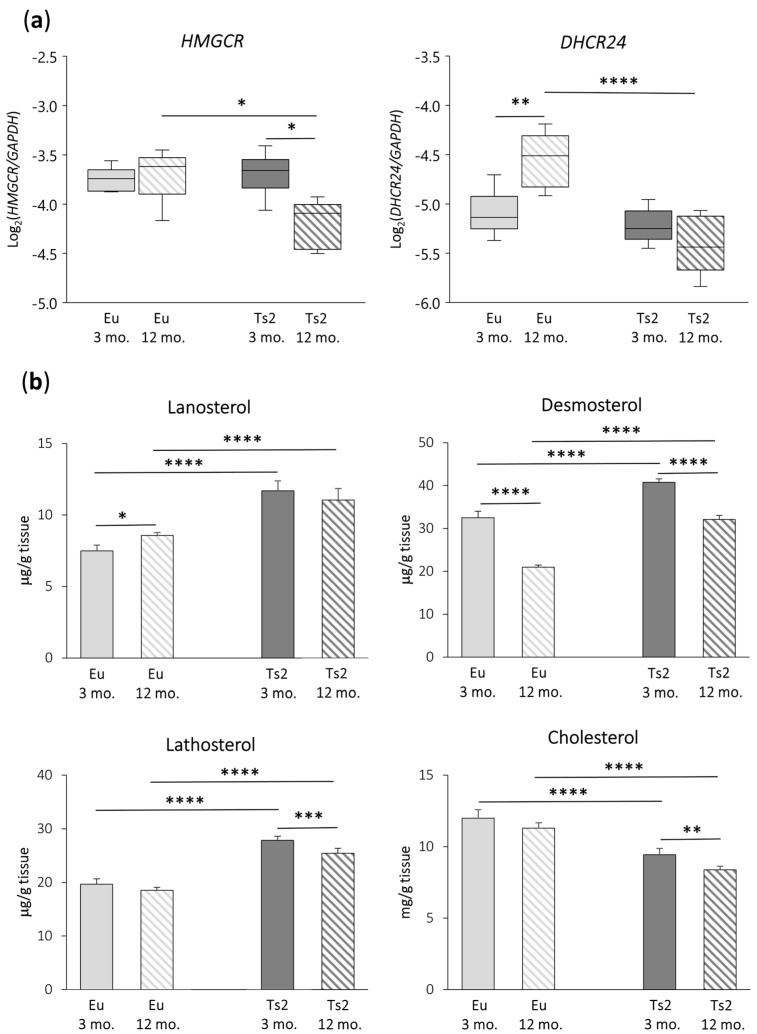
3-hydroxy-3-methylglutaryl-CoA reductase (*HMGCR*) and 24-dehydrocholesterol reductase (*DHCR24*) gene expression analysis and quantification of the levels of cholesterol and its main precursors. (**a**) Expression levels of genes encoding the enzymes HMGCR and DHCR24 were analyzed in the frontal cortices from 3 and 12 mo. Eu and Ts2 mice. Gene expression results were normalized to glyceraldehyde-3-phosphate dehydrogenase (*GAPDH*) expression levels (n = 6/group). **** *p* < 0.0001, ** *p* < 0.01, and * *p* < 0.05; (**b**) Total cholesterol and cholesterol precursors were quantified using gas chromatography–mass spectrometry (GC-MS) in the frontal cortices from 3 and 12 mo. Eu and Ts2 mice. Data are expressed as mean values ± SD (n = 6/group). **** *p* < 0.0001, *** *p* < 0.001, ** *p* < 0.01, and * *p* < 0.05.

To investigate whether cholesterol biosynthesis is deregulated in the frontal cortices of 3 and 12 mo. Ts2 mice, we analyzed the expression of two genes encoding key enzymes involved in cholesterol biosynthesis, namely HMGCR, the rate-limiting enzyme of the mevalonate pathway, and DHCR24, which is relevant in both the Bloch and K-R post-lanosterol multi-step pathways that end in cholesterol formation (Figure 1). As shown in Figure 2a, both *HMGCR* (*p* < 0.05) and *DHCR24* (*p* < 0.0001) appear to be downregulated in 12 mo. Ts2 mice compared to 12 mo. Eu mice. In addition, while in the older Eu mice, *DHCR24* is significantly overexpressed (*p* < 0.01), in the Ts2 mice, it slightly decreases with age. With regard to *HMGCR*, it is significantly reduced (*p* < 0.05) in the Ts2 mice with age, while no changes are observed in the Eu mice.

To understand whether *HMGCR* and *DHCR24* downregulation could affect the production of cholesterol precursors and of cholesterol itself, the sterols have been quantified using GC-MS (Figure 2b). The analysis demonstrated that the levels of cholesterol precursors are significantly higher in Ts2 mice compared to Eu mice of the same age: namely, lanosterol (+56% Ts2 mice 3 mo., +29% Ts2 mice 12 mo., *p* < 0.0001), desmosterol (+26% Ts2 mice 3 mo., +53% Ts2 mice 12 mo., *p* < 0.0001), and lathosterol (+42% Ts2 mice 3 mo., +37% Ts2 mice 12 mo., *p* < 0.0001). On the contrary, the cholesterol levels are lower (−21% Ts2 mice 3 mo., −26% Ts2 mice 12 mo., *p* < 0.0001). Moreover, the production of these molecules diminished more or less significantly in both the Eu and Ts2 mice at 12 mo. of age (desmosterol: −36% Eu mice and −21% Ts2 mice, *p* < 0.0001; lathosterol: −9% Ts2 mice, *p* < 0.001; cholesterol: −11% Ts2 mice, *p* < 0.01), with the exception of lanosterol, which increases in 12 mo. Eu mice (+14%, *p* < 0.05).

Since the synthesis of lathosterol from its precursor lanosterol depends directly on the enzymatic activity of DHCR24, the ratio between lanosterol and lathosterol was calculated in the four populations in order to evaluate the extent of the enzymatic activity, as reported in Table 1. The ratio value is very similar (<1.0) in all the different experimental groups, suggesting that, in the K-R pathway, DHCR24 is equally active in the Eu and Ts2 mice.

Overall, these data point to changes in cholesterol synthesis in the Ts2 mouse brain, both at the level of the expression of genes encoding enzymes of the cholesterol biosynthetic pathway and at the level of formation of cholesterol precursors.

### 3.2. Expression of Genes Encoding the Main Oxysterol-Producing Enzymes and Oxysterol Quantification

**Figure 3:**
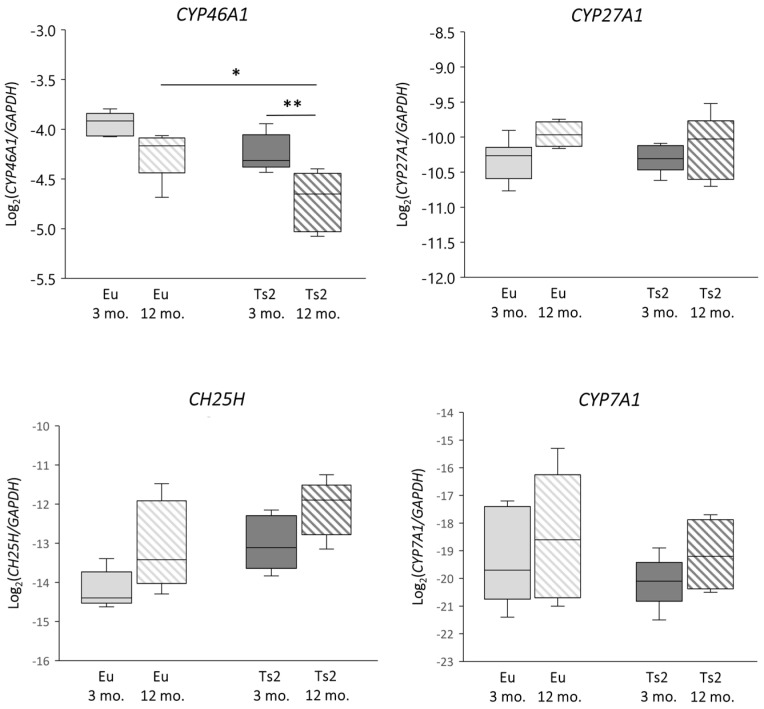
Analysis of the expression levels of genes encoding enzymes involved in cholesterol oxidation. Cholesterol 24-hydroxylase (*CYP46A1*), cholesterol 25-hydroxylase (*CH25H*), cholesterol 27-hydroxylase (*CYP27A1*), and cholesterol 7α-hydroxylase (*CYP7A1*) gene expression levels were analyzed in the frontal cortices of 3 and 12 mo. Eu and Ts2 mice. Gene expression results were normalized to glyceraldehyde-3-phosphate dehydrogenase (*GAPDH*) expression levels (n = 6/group). ** *p* < 0.01 and * *p* < 0.05.

To verify whether abnormalities in sterol biosynthesis in the Ts2 mouse brain could affect the following conversion of cholesterol, we analyzed the expression of the genes encoding the enzymes cholesterol 24-hydroxylase (CYP46A1), cholesterol 25-hydroxylase (CH25H), cholesterol 27-hydroxylase (CYP27A1), and cholesterol 7α-hydroxylase (CYP7A1), as well as the levels of the corresponding oxysterols 24-OHC, 25-OHC, 27-OHC, and 7α-OHC. Specifically, *CYP46A1* expression is reduced in Ts2 compared to Eu mice, but significantly only in the 12 mo. animals (*p* < 0.05). Of note, *CYP46A1* expression also decreases with age in both Eu and Ts2 mice, but the reduction is markedly significant only in Ts2 mice (*p* < 0.01) (Figure 3).

With regard to *CYP7A1*, its expression levels are slightly decreased in the 3 mo. Ts2 compared to the Eu mice, although both populations show an increasing trend with age. Moreover, *CH25H* expression rises in the Ts2 mice, but not significantly compared to the Eu mice. Concerning *CYP27A1*, its expression is similar in both the Ts2 and Eu mice and slightly increases with age (Figure 3).

**Figure 4:**
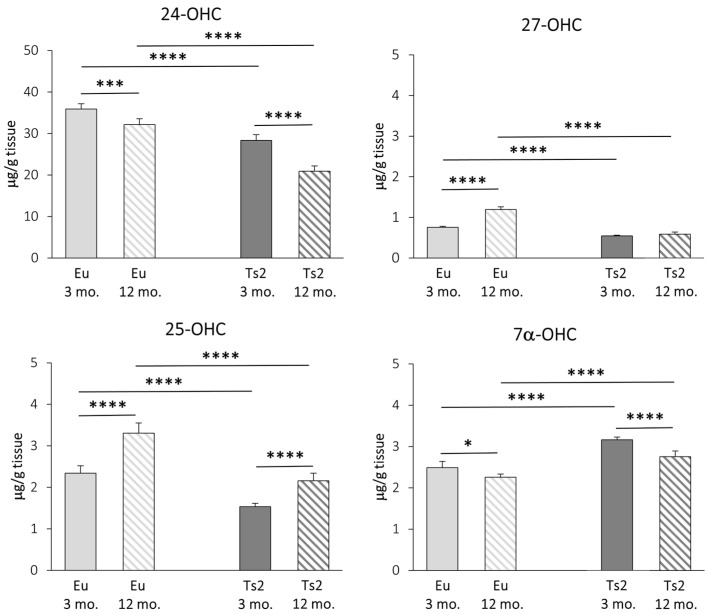
Quantification of the main oxysterols of enzymatic origin. The levels of the oxysterols 24(S)-hydroxycholesterol (24-OHC), 25-hydroxycholesterol (25-OHC), 27-hydroxycholesterol (27-OHC), and 7α-hydroxycholesterol (7α-OHC) in the frontal cortex from 3 and 12 mo. Eu and Ts2 mice were quantified using gas chromatography–mass spectrometry (GC-MS). Data are expressed as mean values ± SD (n = 6/group). **** *p* < 0.0001, *** *p* < 0.001, and * *p* < 0.05.

Concerning the enzymatic oxysterols, their levels in the brain were measured using GC-MS, and, as shown in Figure 4, 24-OHC is the most abundant oxysterol, followed by 25-OHC, 7α-OHC, and 27-OHC as the lowest (24-OHC: 19.51–37.38 µg/g tissue; 25-OHC: 1.38–3.71 µg/g tissue; 7α-OHC: 2.19–3.25 µg/g tissue; 27-OHC: 0.52–1.29 µg/g tissue). The reduction in 24-OHC (−21% Ts2 mice 3 mo., −35% Ts2 mice 12 mo., *p* < 0.0001) (Figure 4) in the Ts2 mouse brain reflects the trend of decreased gene expression levels for the corresponding enzyme (CYP46A1) (Figure 3). Of note, in both the mouse models, 24-OHC levels decrease with age (−10% Eu mice 12 mo., *p* < 0.001; −26% Ts2 mice 12 mo., *p* < 0.0001). Also, 27-OHC shows a significant decrease in the Ts2 mice compared to the Eu mice (−28% Ts2 mice 3 mo., −51% Ts2 mice 12 mo., *p* < 0.0001), as well as 25-OHC (−34% Ts2 mice 3 mo., −35% Ts2 mice 12 mo., *p* < 0.0001), in disagreement with the pattern of the changes in gene expression levels of the enzymes responsible for their synthesis (Figure 3).

In the Ts2 mice, despite *CYP7A1* expression not being enhanced (Figure 3), cholesterol oxidation into 7α-OHC is increased compared to the Eu mice (+27% Ts2 mice 3 mo., +22% Ts2 mice 12 mo., *p* < 0.0001). However, the 7α-OHC levels are shown to decrease with age both in the Eu mice (−9%, *p* < 0.05) and in the Ts2 mice (−13%, *p* < 0.0001) (Figure 4).

Of note, in the Ts2 mice, there is a significant positive correlation between cholesterol and 24-OHC levels (r = 0.7936, *p* < 0.01), a positive correlation between cholesterol and 7α-OHC levels (r = 0.7655, *p* < 0.01), and a marked negative correlation between cholesterol and 25-OHC levels (r = −0.8214, *p* < 0.01). No significant correlation is appreciated between cholesterol and 27-OHC (r = −0.2795) (Figure S1). In the Eu mice, cholesterol positively correlates with 24-OHC (r = 0.7719, *p* < 0.01), while it negatively correlates with 27-OHC, although moderately (r = −0.5872, *p* < 0.05). There are no significant correlations between cholesterol and 7α-OHC or 25-OHC (Figure S2).

Considering all these data, the Ts2 mice seem to suffer also defects in cholesterol catabolism that impact the production of its metabolites.

### 3.3. Evaluation of Oxysterols of Non-Enzymatic Origin

It is well recognized that, under conditions of oxidative stress, cholesterol is prone to autoxidation as a consequence of reactive oxygen species (ROS) attack, leading to the production of different oxysterols [15]. Moreover, as focused on in Section 3.1, Ts2 mice seem to be characterized by an outstanding downregulation of *DHCR24* expression. The DHCR24 enzyme is involved not only in the endogenous cholesterol biosynthesis but also in cell protection against oxidative stress [17,31]. Based on these considerations, we hypothesize that a decrease in *DHCR24* expression, by impairing the antioxidant defense, could exacerbate cholesterol oxidation by ROS.

**Figure 5:**
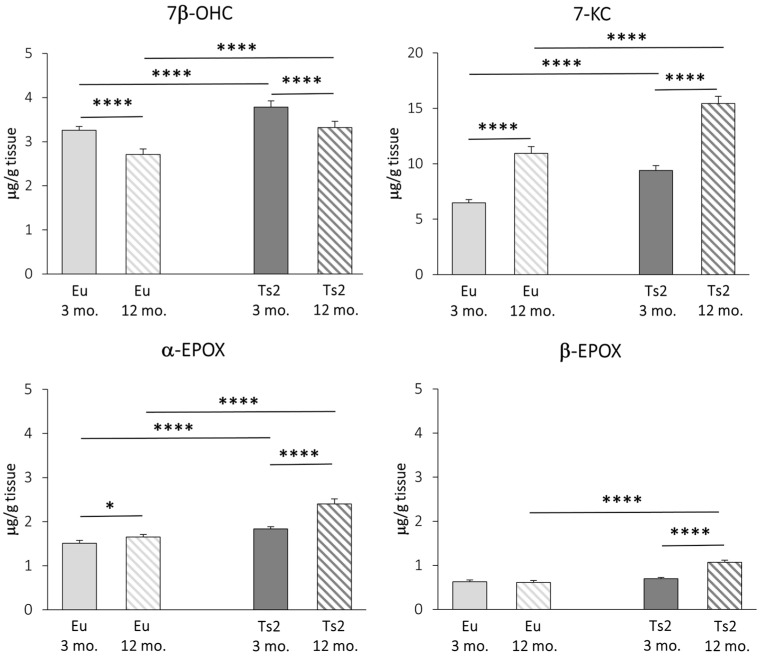
Quantification of the main oxysterols of non-enzymatic origin. The levels of the oxysterols 7β-hydroxycholesterol (7β-OHC), 7-ketocholesterol (7-KC), 5α,6α-epoxycholesterol (α-EPOX), and 5β,6β-epoxycholesterol (β-EPOX) in the frontal cortex from 3 and 12 mo. Eu and Ts2 mice were quantified using gas chromatography-mass spectrometry (GC-MS). Data are expressed as mean values ± SD (n = 6/group). **** *p* < 0.0001 and * *p* < 0.05.

We carried out a GC-MS characterization of the main brain oxysterols of autoxidative origin, namely 7β-OHC, 7-KC, α-EPOX, and β-EPOX. All oxysterols, with the exception of 7β-OHC, increase with age in both Eu and Ts2 mice. However, all these molecules are significantly overproduced in Ts2 mice compared to Eu mice, regardless of age (7β-OHC: +16% Ts2 mice 3 mo., +23% Ts2 mice 12 mo., *p* < 0.0001; 7-KC: +45% Ts2 mice 3 mo., +41% Ts2 mice 12 mo., *p* < 0.0001; α-EPOX: +21% Ts2 mice 3 mo., +46% Ts2 mice 12 mo., *p* < 0.0001), except for β-EPOX, whose levels increase only in the older mice (+74%, *p* < 0.0001) (Figure 5).

These data suggest that, in addition to a decreased expression of *DHCR24*, in Ts2 mice, an exacerbation of oxidative stress already occurs in the young animals.

### 3.4. Analysis of Inflammatory Molecules

It is recognized that DS patients are characterized by aggravation of cerebral inflammation. Therefore, it is likely that Ts2 mice might be characterized by inflammatory cytokine overproduction in the brain. This may occur through: (i) a decrease in DHCR24, which modulates the pathways that underlie neuroinflammation, including type I interferon (IFN) signaling [32,33,34]; (ii) an increase in CH25H, which is responsible for the synthesis of 25-OHC with pro-inflammatory properties [35].

**Figure 6:**
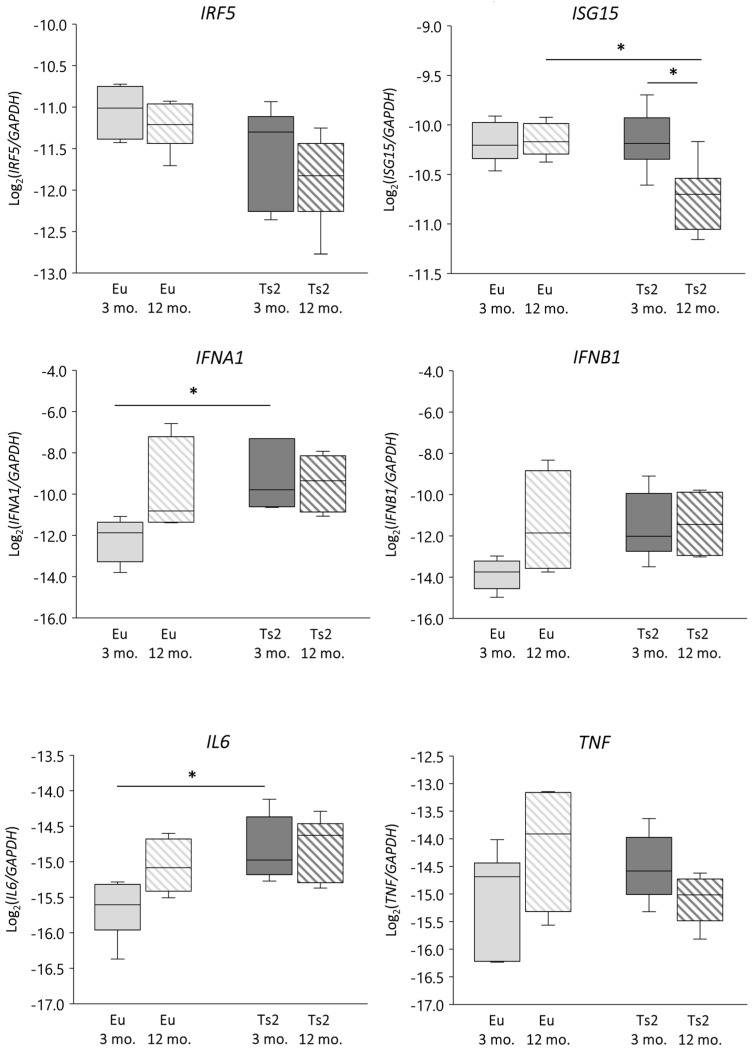
Analysis of the expression levels of genes encoding proteins involved in the inflammatory response. Interferon α (*IFNA1*) and β (*IFNB1*), interleukin 6 (*IL6*), interferon regulatory factor 5 (*IRF5*), IFN-stimulated gene 15 (*ISG15*), and tumor necrosis factor α (*TNF*) gene expression levels were analyzed in frontal cortices from 3 and 12 mo. Eu and Ts2 mice. Gene expression results were normalized to glyceraldehyde-3-phosphate dehydrogenase (*GAPDH*) expression levels (n = 6/group). * *p* < 0.05.

We verified whether *DHCR24* gene lessening was associated with an altered IFN-related response by evaluating not only the expression of *IFNA1* and *IFNB1* but also the expression of IFN regulatory factor 5 (*IRF5*) and IFN-stimulated gene 15 (*ISG15*), both of which play a role in p53/IFN type I signaling [33] and are operative in the context of neuroinflammation [36,37] and the downstream cytokines interleukin 6 (IL-6) and tumor necrosis factor α (TNFα) [34] (Figure 6). In comparison to the Eu animals, in the Ts2 mice *IFNA1* and *IL6* gene expression levels are significantly upregulated at 3 mo. of age (*p* < 0.05), while *IFNB1* is modulated at 3 mo., although not significantly. However, the increase of *IFNA1* and *IL6* expression does not correlate with *DHCR24* expression (Figure S1). There are no significant differences in the mRNA levels of *IRF5* and *TNF* (Figure 6), irrespective of age or presence of trisomy. Also in this case, their expression does not correlate with *DHCR24* expression levels (Figure S1). A significant decrease in *ISG15* expression is observed in the 12 mo. Ts2 mice, both vs. the 12 mo. Eu mice and vs. the 3 mo. Ts2 mice (Figure 6), despite no correlation with *DHCR24* mRNA lessening being observed (Figure S1). Conversely, with regard to the enzymes implied in side-chain cholesterol oxidation, a positive significant correlation exists between *IFNA1* and *CH25H* expression in the Eu but not in the Ts2 mice (r = 0.8195, *p* < 0.01) (Figures S1 and S2).

The evidence suggests that an impairment in IFNα-mediated inflammatory reactions may occur in Ts2 mice, and that CH25H, but not DHCR24, may play a role in these events.

## 4. Discussion

Among the severe clinical outcomes that characterize DS, there is the manifestation of progressive AD-like neurodegeneration, which frequently occurs very early in DS subjects, with clinical presentation between 40 and 50 years [1].

In addition to the trisomy of genes that have been identified to be related to AD etiology, disturbance of brain cholesterol homeostasis has been recognized to take part in AD neurodegeneration. Therefore, it might be implicated in the early onset of AD in DS subjects. As a major component of the plasma membrane and of its well-known microdomains lipid rafts, cholesterol, in coordination with sphingolipids, ensures the correct oligomerization and functioning of several membrane proteins, such as caveolins, thus regulating APP and tau processing and synaptic transmission. Moreover, it regulates neurite outgrowth, it is an essential constituent of myelin sheath, and it is fundamental for axonal growth, synaptic formation, and plasticity [8,38,39,40].

As reported in Figure 1, endogenous cholesterol synthesis is a multi-step process requiring high-energy expenditure that takes place in the endoplasmic reticulum (ER) through two main stages, the “pre-lanosterol stage” and the “post-lanosterol stage”, in which the key enzymes are HMGCR and DHCR24, respectively. DHCR24 regulates cholesterol synthesis through the Bloch and the K-R pathways. In the Bloch pathway, DHCR24 catalyzes the last step of cholesterogenesis by reducing desmosterol, while in the K-R pathway, it acts as the first enzyme of the cascade that gives rise to cholesterol starting from lanosterol through the synthesis of the intermediate lathosterol. Of note, DHCR24 can also shift any Bloch pathway metabolite to the K-R one. Considering the entire anabolic cascade, HMGCR is the only established rate-limiting enzyme that catalyzes an irreversible step, while it is not clear whether the other enzymes participating in the following steps are rate-limiting as well [10,11].

In addition to cholesterol, its oxidation products, namely oxysterols, play important roles in AD progression by promoting or counteracting it. This class of compounds are indeed engaged in cholesterol homeostasis regulation and in many other cellular pathophysiological processes, including inflammation, immunoreactivity, redox balance, and cell death/survival [13]. Brain cholesterol can be metabolized through enzymatic oxidation into oxysterols (Figure 1). The enzymes specifically active in the brain are the hydroxylases CYP46A1, CH25H, and CYP7A1, which convert cholesterol into 24-OHC, 25-OHC, and 7α-OHC, respectively [41,42]. Among these oxysterols, the most abundant is 24-OHC, also known as cerebrosterol, since CYP46A1 is almost exclusively expressed by neurons. It is essential for cholesterol homeostasis regulation because it diffuses across the BBB into the systemic circulation and modulates the expression of genes involved in cholesterol synthesis and efflux from the brain. Also, 25-OHC is operative in cholesterol homeostasis and intracellular transport, and importantly, it acts on the immune system [42]. A minimal amount of cholesterol is converted into 27-OHC by the hydroxylase CYP27A1, but most of it is derived from the peripheral circulation, and its influx in the brain increases under conditions of hypercholesterolemia that alter BBB permeability [43]. In addition, due to the redox imbalance that is typical of AD, cholesterol undergoes non-enzymatic oxidation by overproduced ROS, with the formation of the neurotoxic oxysterols 7-KC, 7β-OHC, α-EPOX, and β-EPOX (Figure 1). These compounds have been implicated in AD onset and aggravation, as strongly supported by several investigations [13,44]. Nevertheless, the possible involvement of cholesterol and its derivatives in DS neuropathology has been poorly explored, although it is considered to be a genetic form of AD. To partially fill this gap, in the present work, we have attempted to uncover whether DS is characterized by perturbations in brain cholesterol metabolism to which the precocious AD-like cerebral complications could be ascribed.

For this purpose, we used Ts2 mice, a well-established DS murine model characterized by a triple copy of a Robertsonian fusion chromosome carrying the distal part of Chr16 and Chr12 [26], to profile cholesterol metabolic processes in the mouse frontal cortex, which is one of the regions affected in the sporadic and familial forms of AD. We analyzed 3 and 12 mo. animals as representative of adult and aged-adult, the latter corresponding to the age at which the AD-like signs appear in DS humans (i.e., about 40 years old) [45,46].

An expression analysis of the genes encoding HMGCR and DHCR24, the key enzymes in the pre- and post-lanosterol stages of cholesterogenesis, respectively, evidenced a down-regulation of the transcription of both the genes in the frontal cortex of the older Ts2 mice compared to the Eu mice. Moreover, *HMGCR* gene expression decreased in the Ts2 mice with aging (Figure 2a). Consistent with this, cholesterol levels also decreased in the Ts2 mice compared to the Eu mice. Moreover, cholesterol synthesis declined with age in the Ts2 mice, which could be a consequence of cell function worsening or cell death associated with sustained neurodegeneration (Figure 2b). Overall, this evidence highlights that an impairment in brain cholesterol synthesis likely occurs in DS.

Despite the decrease in *HMGCR* and *DHCR24* gene expression, the amount of the cholesterol precursors lanosterol, desmosterol, and lathosterol are enhanced in the Ts2 mouse brain compared to the Eu mice, although they showed a decline with age (Figure 2b). This could be the result of the different actions of all the cell types involved. In this connection, in physiological conditions, developing brain neurons significantly participate in cholesterol generation, more than astrocytes, and all cell types employ the Bloch pathway. The situation is different in the postnatal brain when cholesterol requirements are almost completely provided by glial cells, mainly astrocytes, again through execution of the Bloch cascade, while neurons contribute minimally, following the K-R pathway. Thus, cerebral cholesterogenesis, throughout the entire life cycle, preferentially relies on the Bloch pathway, in which lanosterol is mostly converted into desmosterol and then into cholesterol by DHCR24. Of note, although DHCR24 can theoretically intervene at any stage to switch the Bloch to the K-R cascade, under physiological conditions, it happens in a very limited manner, and lathosterol production is low [12,47].

It is likely that under pathological conditions, such as DS, the equilibrium in the brain between the Bloch and the K-R synthesis could be altered depending on the cell populations. We presume that the Bloch arm importantly suffers from the DHCR24 deficiency observed in Ts2 mouse brain; indeed, desmosterol cannot be efficiently transformed into cholesterol by astrocytes and significantly accumulates, as shown in Figure 2b. Of note, the excess of desmosterol is detrimental, as proven in individuals affected by desmosterolosis, a developmental disorder due to the defective DHCR24 gene. These individuals are affected by organ malformations, including the brain, since desmosterol replaces cholesterol in cell membranes, in particular in lipid rafts, disturbing the signaling of several proteins [17].

Surprisingly, in addition to desmosterol loading, in the Ts2 mice, an increase in lathosterol was observed compared to the Eu mice (Figure 2b). Since lathosterol formation in the K-R arm depends on DHCR24 activity, its increment is probably due to an increased activity of neuronal DHCR24 in Ts2 mice, although its *DHCR24* expression throughout the whole tissue is decreased (Figure 2a). To test this hypothesis, the ratio between lanosterol and lathosterol levels was calculated for all the groups (Table 1). The ratio value, less than 1.0, is very similar in all brain samples, suggesting that in Ts2 mice, DHCR24 is as active as in Eu mice in converting lanosterol into lathosterol. On this basis, we hypothesize that, in Ts2 mice, astrocytes are unable to provide the right cholesterol amount via the Bloch pathway, and neurons try to compensate for this lack through the K-R pathway. Nevertheless, this compensatory mechanism seems to fail, leading to lathosterol accumulation and a consequent decrease in cholesterol content (Figure 2b). This could be due to the presence of defective lathosterol-downstream enzymes, constraining its further metabolization. Moreover, the entire process could be interrupted as too expensive in terms of energy consumption. Neurons are indeed pivotal for synaptic signal transmission, a fundamental process with a very high energy requirement, and they dramatically pay for ATP impairment due to the mitochondrial dysfunctions associated with DS [48]. In the Ts2 mouse brain, an accumulation of lanosterol, despite *HMGCR* downregulation, was also observed, presumably as a consequence of both Bloch and K-R inefficiency, which limits its further conversion (Figure 2b).

Notably, some of the current results resemble the data reported by our group in a previous study focused on cholesterol homeostasis in *ApoE4* astrocytes. Indeed, we have demonstrated that Ts2 mice, in comparison to Eu mice, show *HMGCR*, *DHCR24*, and cholesterol trends similar to those already observed in *ApoE4* astrocytes compared to *ApoE3* [29]. However, we must take into account that the two experimental models are different. The findings herein reported are indeed representative of changes that arose in the brain over 3 or 12 mo. affecting all cell populations, not just astrocytes. This suggests that derangements in brain cholesterol synthesis could be ascribed to defective gene transcription in astrocytes, which might underpin AD-like manifestations in young DS people carrying *ApoE4* [21]. The most remarkable evidence observed in both the Ts2 brains and *ApoE4* astrocytes is the down-regulation of *DHCR24* gene expression. As demonstrated by Greeve and colleagues, the *DHCR24* gene is downregulated in AD-vulnerable regions, and the consequent reduction in the levels of this enzyme might favor oxidative stress and Aβ toxicity, thus inducing cell death [16]. Later, in addition to its metabolic role in cholesterogenesis, other properties have been attributed to DHCR24, such as the anti-inflammatory activity that helps to counteract AD development. Thus, *DHCR24* downregulation in the brain might represent a crucial event on which new research effort should focus [11,17,18].

The limited expression of *HMGCR* and *DHCR24* and lower cholesterol content in our Ts2 mouse model and *ApoE4* astrocytes [29] imply meaningful issues about the pharmacological treatment of AD-like neurodegeneration with the use of lipid lowering agents, in particular statins which are HMGCR inhibitors. Although the effects of statins are mainly at the systemic level, these drugs, in particular the lipophilic simvastatin which appears the most effective at crossing the BBB, are proposed as neuroprotective in the early stages of AD thanks to their hypocholesterolemic activity, and antioxidant and anti-inflammatory properties [49,50,51]. Statins can indeed inhibit neuroinflammation and associated neurodegenerative complications through inhibition of microglia and astrocyte activation, and of the release of pro-inflammatory cytokines [52,53]. Moreover, it has been shown that statin pharmacotherapy could be mainly beneficial for AD patients with dyslipidemia [54], for instance in patients with homozygous *ApoE4* genotype [55].

However, the in vitro and clinical evidence of their efficacy is controversial, and is principally ascribed to their potential cytotoxicity [56]. For instance, high-doses of lipophilic statins have been shown to cause transient and reversible cognitive dysfunction, probably due to an excessive suppression of brain cholesterol biosynthesis that alters neuronal membrane composition and leads to deficits in neurotransmission [54]. In this connection, it is important to keep in mind that the cerebral effects of statins could also be due to other mechanisms, independently of their cholesterol-lowering effects [54].

The significant decrease in *CYP46A1* in Ts2 older mice (Figure 3) encoding the principal enzyme engaged by neurons to oxidize brain cholesterol into 24-OHC suggests that the cholesterol catabolic phase is also affected, likely as a consequence of neuron loss occurring during progressive neurodegeneration. In support of this evidence, we also observed a decrease in the CYP46A1 enzyme in post-mortem human AD brains, classified by a Braak staging system of neurofibrillary pathology [9]. *CYP46A1* downregulation was also found in ApoE4 astrocytes, highlighting another possible common feature between DS and AD [29].

Of note, since *CYP46A1*, *HMGCR*, and *DHCR24* are not located on Hsa21 or on its syntenic Mmu16 [57], it can be hypothesized that trisomy compromises the expression/activity of proteins of auxiliary pathways involved in cholesterol metabolism; a very complex and finely tuned process. This consideration is supported by the “amplified developmental instability hypothesis”, according to which DS does not exclusively affect specific genes on the triplicated chromosome but rather different sets of genes, which leads to genetic instability or dyshomeostasis [58]. Epigenetic modulation or the action of non-coding genes like miRNAs could also interfere [59,60].

Of note, cell membrane derangements due to alterations in its cholesterol content have been observed in several neurological diseases and have been extensively investigated in AD, which has recently been identified as a plasma membrane disorder [38]. In addition, mitochondria-associated ER membranes (MAMs) have recently gained attention in the study of AD pathogenesis. MAM is a lipid raft-like subdomain of the ER that, by connecting with its counterpart on mitochondria, drives several processes involved in brain cholesterol metabolism and trafficking, including cholesterol esterification, the interaction of lipoprotein receptors with ApoE, and the production of ATP necessary for cholesterol biosynthesis by mitochondria [61,62]. Although little is known about membrane cholesterol equilibrium in DS, it cannot be excluded that it is also directly or indirectly perturbed by aneuploidy.

In addition to cholesterol biosynthesis, the production of its oxidized derivatives, named oxysterols, was analyzed. With regard to the oxysterols of enzymatic origin 24-OHC, 25-OHC, 27-OHC, and 7α-OHC, the pattern of their levels in Ts2 mice is variable (Figure 4) and in some cases not consistent with the expression of the corresponding hydroxylases (Figure 3). Among the analyzed oxysterols, 24-OHC levels are markedly decreased in Ts2 mice, mainly in older animals, in parallel with *CYP46A1* expression (Figure 3 and Figure 4). Of note, the amount of 24-OHC exhibits a moderate but significant positive correlation with cholesterol (Figure S1). A growing body of literature highlights the physiological role of 24-OHC as a signaling molecule that is crucial for brain functions. Indeed, it is a positive allosteric modulator of the N-methyl-D-aspartate receptors (NMDARs), which are crucial for synaptic plasticity and learning and whose sensitization is orchestrated in membrane rafts [40]. Moreover, it also appears to exert protective effects against amyloid plaque formation [63] and pTau [64,65]. In consideration of these neuroprotective properties, the lower amounts of 24-OHC in the brains observed in our experimental model (Figure 4) could explain the worsening of cerebral performance in DS individuals.

With regard to 27-OHC, its decline in the Ts2 brains (Figure 4) may be due to its marked conversion into 7α-hydroxy-3-oxo-4-cholestenoic acid, a cholesterol derivative that easily crosses the BBB to reach the bloodstream [13]. A minimally significant correlation between 27-OHC and brain cholesterol levels in the Eu mice (Figure S2) was observed, but no correlation was found in the Ts2 mice (Figure S1).

The oxysterol 25-OHC, which is enzymatically produced by CH25H and synthesized by microglia to trigger immune responses, is reduced in Ts2 mice compared to Eu mice (Figure 4) despite the slight increase in *CH25H* expression. Despite this, we observed a significant 25-OHC increase in the older Ts2 and Eu mice. Because 25-OHC is also an oxysterol of autoxidative origin, this could underline the presence of chronic inflammation and oxidative stress, which are normally observed in cell aging (Figure 3). Notably, previous studies have instead reported an increase in 25-OHC production in neuroinflammatory disorders such as AD, in particular in the presence of the *ApoE4* genotype [66] as well as in ApoE4 astrocytes [29].

Concerning 7α-OHC, an oxysterol originating through both enzymatic and non-enzymatic mechanisms, its autoxidation likely accounts for its presence as a major content in the Ts2 brain (Figure 4), since the expression of the gene encoding the enzyme CYP7A1 appears to be slightly reduced in Ts2 mice and not significantly different compared to Eu mice (Figure 3). A marked positive correlation between 7α-OHC and cholesterol levels in the Ts2 mice indicates that the reduction in 7α-OHC levels with age may depend on reduced cholesterol availability (Figure S1).

Of note, under physiological conditions, chain-hydroxylated oxysterols, such as 24-, 25-, and 27-OHC, act as feedback regulators of cholesterol intracellular content. In the case of cholesterol deficiency, and consequently of low oxysterol levels, their lessening activates the transcription factors sterol responsive element binding proteins (SREBPs), which in turn induce *HMGCR* and *DHCR24* expression, thus restoring cholesterol synthesis [67]. In this connection, cholesterol sensing and homeostasis are afforded by side-chain oxysterols, since they can modify membrane structure through insertion into the lipid bilayer [68]. In contrast, in our model, the decrease in the above oxysterols does not lead to increased expression of *HMGCR* and *DHCR24*, suggesting that this compensatory mechanism is dysfunctional in Ts2 mice and that severe membrane derangements might participate in its dysregulation.

A remarkable point is the highly significant accumulation in the brain of both 3 and 12 mo. Ts2 mice of cholesterol autoxidation products, i.e., 7β-OHC, 7-KC, α- and β-EPOX (Figure 5), as well as 7α-OHC (Figure 4). This evidence is consistent with the assumption that oxidative processes are already exacerbated in the DS brain at early ages, representing a link between DS and AD etiology [69]. We have previously quantified non-enzymatic oxysterols in post-mortem human AD brains and found that their quantities increased with the severity of the disease, alongside inflammatory parameters [9]. Autoxidative oxysterols exert several harmful and cytotoxic activities that are entailed in the activation of inflammatory pathways, mitochondrial and endoplasmic reticulum stress, apoptosis, necrosis, and genotoxicity [70]. Moreover, they promote ROS production, establishing a noxious loop that further sustains oxidative damage [71]. It is reasonable that many of oxysterol activities depend on alterations in biomembrane physico-chemical properties, since all these molecules can be inserted into the lipid bilayer, affecting vital cell processes [72] and favoring neurodegeneration [73]. For all these reasons, the autoxidative oxysterols could be considered to be pivotal intermediates in the oxidative stress-induced AD-like brain injury observed in DS.

In this study, the impairment of *DHCR24* expression observed in the Ts2 mice (Figure 2a) could explain the intensification of the oxidative insult. Indeed, DHCR24 per se is a hydrogen peroxide (H_2_O_2_) scavenger; therefore, its deficiency weakens the cell capability to counteract the ROS overproduction involved in cholesterol oxidation [31]. This aspect could be critical in DS, since the superoxide dismutase (SOD1) gene is located on the triplicated chromosome and its upregulation leads to an imbalance in the cell antioxidant system and accumulation of H_2_O_2_ [69]. Additionally, DHCR24 has been indicated as a key sensor and regulator of the cell redox status by suppressing the acetylation of the tumor suppressor p53 and, in turn, by favoring the inhibition of its transcriptional activity via mouse double minute-2 (Mdm2). This signaling is thought to control intracellular ROS levels, and its functioning could be compromised in the case of DHCR24 loss [74,75]. Of note, this observation is consistent with a previous analysis of the frontal cortex of DS humans with AD pathology and of DS mice; in these brains, lower levels of the p53/Mdm2 complex and a greater extent of p53 activation associated with a pro-apoptotic phenotype were found [76].

The p53/Mdm2 complex is also implied in the type I IFNα and IFNβ pathways [33], which were recognized to participate in chronic brain inflammation in neurodegenerative diseases, including AD [34]. Of note, the genes coding for IFN receptors are located on the Hsa21 in humans, as well as on the Mmu16 syntenic regions in Ts2 mice; therefore, an amplification of the IFN inflammatory signaling often occurs in DS brains, similarly to what happens in AD [4,6,34]. In light of this, DHCR24, by acting on the p53/Mdm2, might affect the type I IFN-dependent response in the DS brain.

Our data suggest a possible enhancement of the inflammatory response in Ts2 mice, since both *IFNA1* and its downstream *IL6* appeared to be overexpressed (Figure 6). Since the expression levels of the genes encoding ISG15 and IRF5, two mediators of the p53/type I IFN pathway implicated in neuroinflammation [33,36,37], do not show any correlation with *DHCR24* expression (Figures S1 and S2), we hypothesize that DHCR24/p53/IFNs signaling does not underlie cytokine expression. Presumably, other effectors of p53 signaling including toll-like receptors (TLRs) [33] are responsible for *IFNA1* and *IL6* expression in our DS mouse model. Interestingly, TLR4 is activated in lipid rafts; thus, it cannot be ruled out that 25-OHC can modulate TLR signaling as a biomembrane sterol component. This is consistent with the “Inflammaraft hypothesis” that was recently proposed to explain glia cell activation in neuroinflammation [77]. Moreover, it cannot be excluded that cytokine expression levels are indirectly affected by defective genetic and epigenetic features due to trisomy. The increased availability of IFNα could further amplify the inflammatory events caused by IFN receptor overexpression-induced by trisomy, as corroborated by the *IL6* enhancement observed in the Ts2 mice (Figure 6).

Interestingly, we have found a significant positive correlation between *IFNA1* and *CH25H* gene levels in Eu mice but not in Ts2 mice. Robertson and colleagues observed that type I IFNs are able to modulate CH25H, which leads to the production of the immune-modulator 25-OHC, an alternative way to orchestrate the brain immune and inflammatory system [78]. The observed correlation between *IFNA1* and *CH25H* genes in Eu mice could prove to be a correct execution of this mechanism. Conversely, this mechanism might be affected in Ts2 animals due to the lower levels of 25-OHC. Notably, regular 25-OHC production can be protective for the brain, not only for the immune-related activity of this oxysterol, but also because it prevents cholesterol accumulation, reduces neuronal loss, and modulates synaptic transmission and myelinization [42,67]. Thus, anomalies in 25-OHC-related responses could impact brain functionality and contribute to cognitive deficiencies in DS subjects. Moreover, non-enzymatic oxysterols, which accumulate in the Ts2 brain, could also contribute to cytokine expression thanks to their pro-inflammatory action.

## 5. Conclusions

This is the first investigation aimed at shedding light on cholesterol metabolism in the brain of a DS mouse model and at understanding the possible consequences in terms of oxidative and inflammatory damage.

The most meaningful findings we uncovered in the Ts2 mice are as follows: (i) altered expression of genes encoding enzymes that are crucial for cholesterogenesis (*HMGCR*, *DHCR24*) and for cholesterol processing (*CYP46A1*); (ii) decreased levels of cholesterol and oxysterols of enzymatic origin involved in brain cholesterol homeostasis (24-OHC, 25-OHC, and 27-OHC); (iii) increased levels of cholesterol autoxidation products (7-KC, 7β-OHC, α-EPOX, and β-EPOX), markers of brain redox status impairment that could be ascribed, at least in part, to the observed DHCR24 decline; (iv) increased expression of genes encoding pro-inflammatory cytokines, possibly dependent on the dysfunctional IFNα/CH25H/25-OHC pathway and/or on pro-inflammatory cholesterol oxidation products.

Because our research is a pilot study, some limitations need to be pointed out. First of all, considering the complexity of brain cholesterol metabolism, we cannot state whether DS trisomy per se is responsible for the aberrant expression of the cholesterol-related genes observed in the Ts2 mice. It cannot be excluded that the abnormalities in cholesterol metabolism could be the consequence of the cerebral inflammation or oxidative stress observed in DS. It would be useful to evaluate the effects of trisomy on other enzymes participating in cholesterogenesis. Moreover, in addition to the animal models, in vitro experiments would help to better clarify, in both neurons and astrocytes, the pathways involved in brain cholesterol dysmetabolism and neuroinflammation in DS mice.

Taking into account the prominent role played by cholesterol and its derivatives in the organization of membrane lipid bilayers, additional information could be provided by investigating whether cell membrane properties are upset in DS, as well as the relative consequences in terms of brain lipid homeostasis and lipid–protein interactions responsible for inflammation and oxidative insults.

Despite these considerations, our data highlight that brain cholesterol metabolism is dysregulated in DS and that it could be relevant in the exacerbation of the oxidative and inflammatory processes. Since these events are also present in the AD brain, they may be involved in the precocious manifestation of AD-like neuropathology in DS patients. Moreover, drugs targeting the diverse processes that are altered in brain cholesterol homeostasis may offer an alternative and valuable strategy to counteract neuroinflammation and neurodegeneration.

## Figures and Tables

**Table 1:** Extent of lanosterol conversion into lathosterol by DHCR24.

Animal Model	Lanosterol (µg/g Tissue)	Lathosterol (µg/g Tissue)	Lanosterol/Lathosterol Ratio
Eu 3 mo.	7.49	19.67	0.38
Eu 12 mo.	8.57	18.54	0.41
Ts2 3 mo.	11.70	27.84	0.42
Ts2 12 mo.	11.05	25.43	0.43

## Data Availability

The original contributions presented in the study are included in the article/Supplementary Materials. Further inquiries can be directed to the corresponding author.

## Supplementary Materials

The following supporting information can be downloaded at: https://www.mdpi.com/article/10.3390/antiox13040435/s1, Figure S1: Correlation analyses of data obtained from Ts2 mice; Figure S2: Correlation analyses of data obtained from Eu mice. Pearson correlation coefficient (r) and *p*-value (*p*) are reported in the graphs. 24-OHC, 24(S)-hydroxycholesterol; 25-OHC, 25-hydroxycholesterol; 27-OHC, 27-hydroxycholesterol; 7α-OHC, 7α-hydroxycholesterol; *CH25H*, cholesterol 25-hydroxylase; *CYP46A1*, cholesterol 24-hydroxylase; *CYP27A1*, cholesterol 27-hydroxylase; *DHCR24*, 24-dehydrocholesterol reductase; *IFN*, interferon; *IL*, interleukin; *IRF5*, interferon regulatory factor 5; *ISG*, IFN-stimulated gene; *TNF*, tumor necrosis factor.
