# Supplementary figures and images for: Altered Brain Cholesterol Machinery in a Down Syndrome Mouse Model: A Possible Common Feature with Alzheimer’s Disease
Source: Antioxidants (Basel). 2024 Apr 3;13(4):435. doi: 10.3390/antiox13040435 (PMC11047305; doi:10.3390/antiox13040435)

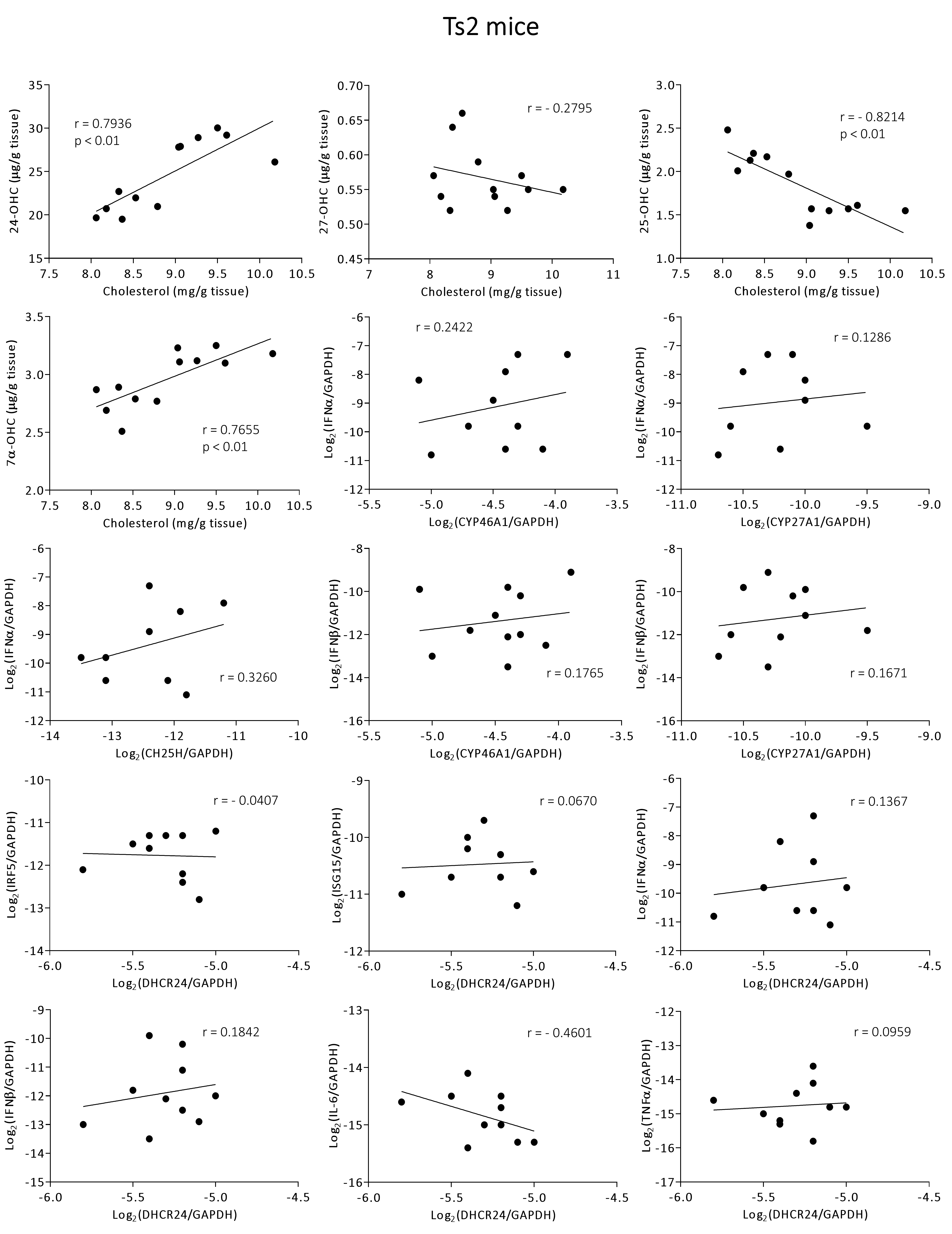

Supplement: Supplementary file 1 [file antioxidants-13-00435-s001.zip › Supplementary Figures/Figure_S1.tif]

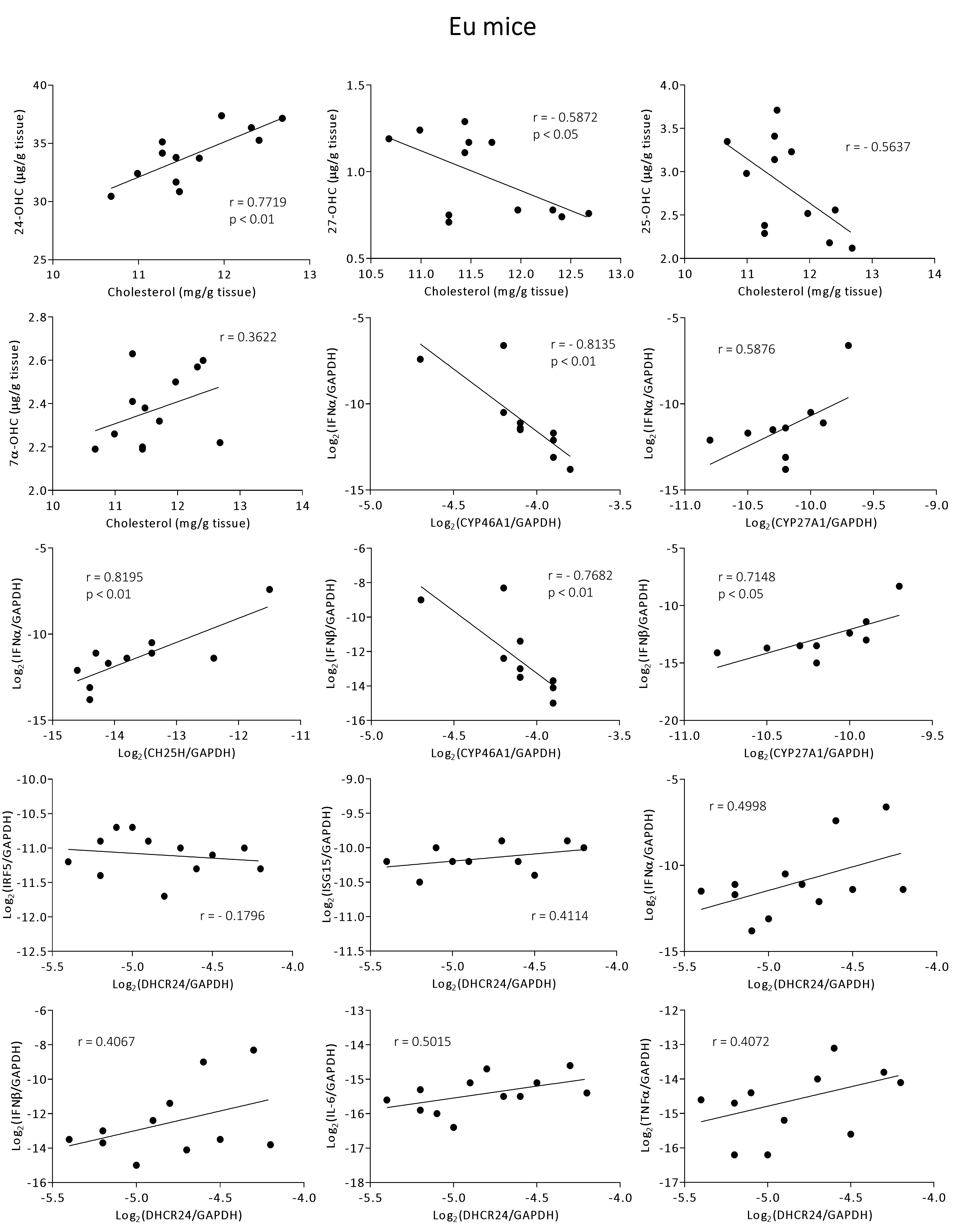

Supplement: Supplementary file 1 [file antioxidants-13-00435-s001.zip › Supplementary Figures/Figure_S2.tif]
